# A 15-Year-Old With Tetralogy of Fallot With Pulmonic Atresia, Multiple Aorticopulmonary Collateral Arteries and Bilateral Peripheral Pulmonary Stenosis

**DOI:** 10.7759/cureus.11151

**Published:** 2020-10-25

**Authors:** Julia D Katz, Sivan Elkarat

**Affiliations:** 1 Pediatric Cardiology, Sackler Faculty of Medicine, Tel Aviv University, Tel Aviv, ISR; 2 Pediatric Cardiology, Wolfson Medical Center, Holon, ISR

**Keywords:** cardiology, tof, tetralogyoffallot, pediatrics

## Abstract

Tetralogy of Fallot (TOF) is a congenital disorder comprised of four heart defects. It is considered a critical condition that requires surgical repair. TOF may be complicated by pulmonary valve atresia (PA) and the development of major aorticopulmonary collateral arteries (MAPCA). A 15-year-old girl from Jimma, Ethiopia, was admitted to the pediatric cardiology unit through the "Save a Child's Heart" organization. She was diagnosed at the age of four years with TOF but had not yet received treatment for the condition. Echocardiography prompted a diagnosis of TOF with PA and MAPCA. It is recommended that TOF be treated shortly after birth, thus observing this condition in 15-year-old highlights the complications that may accompany delayed repair. This report details the course of diagnosis and treatment for this patient.

## Introduction

Tetralogy of Fallot (TOF) is a congenital disorder comprised of four heart defects. The four pathologies characterizing TOF are a ventricular septal defect (VSD), right ventricular hypertrophy, pulmonary stenosis, and an "overriding aorta," which describes an enlarged aortic valve opening from both ventricles [1]. Roughly 1% of newborns have some congenital heart defect, 10% of which being TOF [2]. This defect reduces the amount of available oxygen in the blood, leading to cyanosis. This cyanosis is typically not apparent at birth. However, under conditions that rapidly decrease the amount of available oxygen, such as crying, feeding, or agitation, the infant may become cyanotic. These episodes are known as Tet spells, in which the infant's mouth, skin or nails suddenly appears blue. Complications of TOF may include an increased risk of infection, arrhythmia, delayed development, and direct consequences of hypoxemia, such as dizziness, fainting, or seizures. As such, this condition is considered critical and often requires surgical repair shortly after birth. This condition may be diagnosed in utero by ultrasonography [3], at birth with a pulse oximetry [4], or during an early Tet spell [1].

Tetralogy of Fallot may be further complicated by pulmonary valve atresia (PA). In the case of PA, normal antegrade flow from the right ventricle to the pulmonary artery is absent. This results in the development of major aorticopulmonary collateral arteries (MAPCA), which are characterized by vessels arising directly from the aorta or its branches [5]. In normal circumstances, these arteries are present in utero to connect the systemic and pulmonary circulations, and they regress as the pulmonary arterial system develops. A failure of the pulmonary system to mature encourages the further development of collateral circulation to provide adequate blood flow to the lungs [6]. TOF with PA and MAPCAs is considered the most extreme form of TOF and may be seen in 20-25% of patients with TOF [7]. 

MAPCAs as a compensatory mechanism is problematic because they form in unpredictable patterns, perfusing the lungs inconsistently with the excessive or inadequate flow in different regions. These vessels also have an increased risk of becoming narrow or stenotic over time [8]. The size of the vessels and the degree of stenosis of the MAPCAs will determine the severity of the presentation of the patient [6]. Due to the variability of MAPCA vessels between cases, management of the condition may be complicated. Surgical repair is required and has a good prognosis, with a post-repair survival rate of 92.5% at 10 years of age. Reparative surgery is recommended soon after birth, and the prognosis declines with chromosomal abnormalities, older age, additional collaterals and higher post-repair right ventricular pressure [7]. Failure to repair this condition surgically in the first year may reduce the survival rate to as low as 50%, and the survival rate without surgery may be reduced to 8% at 10 years of age [9]. Our case presentation highlights a patient who received treatment for TOF with PA/MAPCAs at 15 years of age.

## Case presentation

History

A 15-year-old girl from Jimma, Ethiopia was admitted to the pediatric cardiology unit at Wolfson Medical Center through the "Save a Child's Heart" organization on May 15th, 2018. She was diagnosed with TOF at the age of four years, with diagnosis prompted by fatigue and the presence of Tet spells. The patient was within appropriate growth and developmental parameters for her age and attended school. She reports difficulties with moderate exercise. Upon admission, the patient was not taking any medications, had no known allergies or notable family history.

Physical examination

Physical examination showed general well-being as the patient appeared comfortable without respiratory distress or dysmorphism. The patient was cyanotic and had clubbing of the digits. Chest examination demonstrated a right heave. Heart auscultation revealed a normal S1 and S2 heart sound, and a grade 2/6 soft continuous murmur at base of the heart radiating to the back. Lungs were clear to auscultation, and the liver was not palpable. The observable cyanosis and clubbing and the audible murmur on auscultation prompted the differential diagnoses. These included cyanotic congenital heart disease and a persistent ductus arteriosus, causing a right to left shunt.

Imaging

Electrocardiography (ECG) displayed increased right atrial and ventricular forces, with normal T waves and ST segments, and an incomplete right bundle branch block pattern. Echocardiography revealed normal systemic and pulmonary veins with an intact atrial septum. The right atrium was enlarged, and the left atrium was not. The tricuspid valve appeared normal with normal diastolic flow and no regurgitation. The annulus of the tricuspid valve was measured to be 2.2cm.

**Figure 1:**
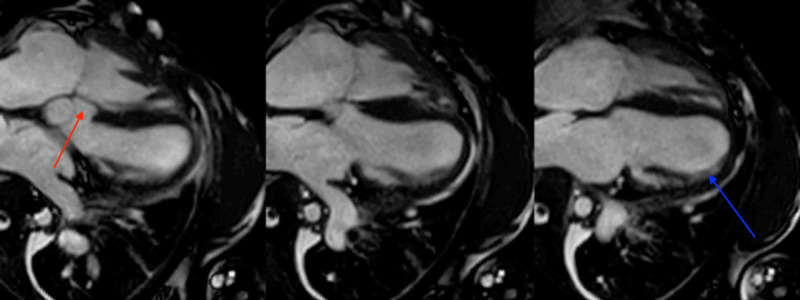
Ventricular Septal Defect MRI imaging displays a defect in the interventricular septum (red arrow). Imaging also shows evidence of right ventricle (RV) enlargement (blue arrow).

The mitral valve appeared normal with normal diastolic flow and no regurgitation. The annulus of the mitral valve was measured to be 2.5 cm. Imaging of the left ventricle did not show enlargement or hypertrophy, with good function. Left ventricular internal dimension was measured as 3.7 mm at end-diastole and 2.7 mm at end-systole. Left ventricular posterior wall thickness at end-diastole was measured to be 0.8 mm. The ratio of the left atrial dimension to the aortic annulus dimension was measured to be 0.6. Imaging displayed right ventricle (RV) enlargement and hypertrophy, with good function. Ventricular septum contained a large, misaligned ventricular septal defect (VSD) with an overriding aorta and a right to left shunt (Figure 1). Interventricular septal thickness at end-diastole was measured to be 0.8mm.

The aortic valve appeared structurally normal, with mild regurgitation and no acceleration to flow. The coronary arteries were normal in origin and proximal course. The ascending aorta was normal, and aortic arch showed no coarctation. The pulmonic valve was atretic. Right pulmonary artery (RPA) was measured to be 9 mm in diameter. The left pulmonary artery (LPA) was not demonstrated. Multiple small aorticopulmonary collateral arteries were present. Imaging showed no evident pleural or pericardial effusion. The systolic filling fraction was 30%. Chest radiology revealed a normal size cardiac silhouette. 

Diagnosis

A diagnosis of tetralogy of Fallot with pulmonic atresia and multiple aorticopulmonary collateral arteries was made.

Treatment

A diagnostic cardiac catheterization was performed on day +14 to delineate true pulmonary arteries and aorto-pulmonary collaterals better. Injections demonstrated major aorto-pulmonary collaterals arising from descending aorta, supplying both the RPA and LPA with distal narrowing. The measurements made during this catheterization were used to calculate a McGoon index of 1.63 and a Nakata index of 152.

Day +69: Treatment began with heart surgery accessed via mid sternotomy. The VSD was closed with a fenestrated VSD patch. A bicuspid pulmonic valve was created. An anastomosis was created from the right upper long collateral artery to the RPA. Following this surgery, the patient was hemodynamically unstable and was unable to be weaned off the ventilator. 

Day +74: The patient underwent interventional cardiac catheterization, during which two collaterals emerging from the descending aorta were closed. Due to the patient's hemodynamic instability following the catheterization, an atrial septal defect (ASD) and VSD were created via surgery, following which she required extracorporeal membrane oxygenation (ECMO) support.

Day +80: Cardiac catheterization was used to place five stents in the LPA and its branches, as well as the removal of RPA thrombus and an umbrella device.

Day +82: During an attempt to wean the patient off ECMO, significant bleeding occurred from the LPA. The LPA was sutured, and ECMO support was re-instituted.

Day +88: Coronary computed tomography angiogram (CTA) revealed multiple filling defects in the pulmonary arteries, superior vena cava and inferior vena cava. CTA also demonstrated ground-glass infiltrates, pneumothorax and small pneumomediastinum. 

Day +93: Patient was successfully weaned off ECMO, and the chest cavity remained open.

Day +97: Chest cavity was closed.

Day +135: Diagnostic catheterization displayed shunt fraction (QP: QS) of 1.05, a large VSD with a left to right shunt, a 6 mm long proximal narrowing of the right upper pulmonary artery, and stent narrowing of the left lower pulmonary artery. Pulmonary blood pressure was 83% of systemic blood pressure. Pulmonary resistance was calculated to be 0.99 Wood units. Consequently, the patient began treatment with Levosimendan and Milrinone. 

Day +175: Following failed attempts to be weaned off the ventilator and repeated re-intubations due to respiratory failure, the patient received a tracheostomy, with gradual weaning off the ventilator to oxygen support as needed. 

**Figure 2:**
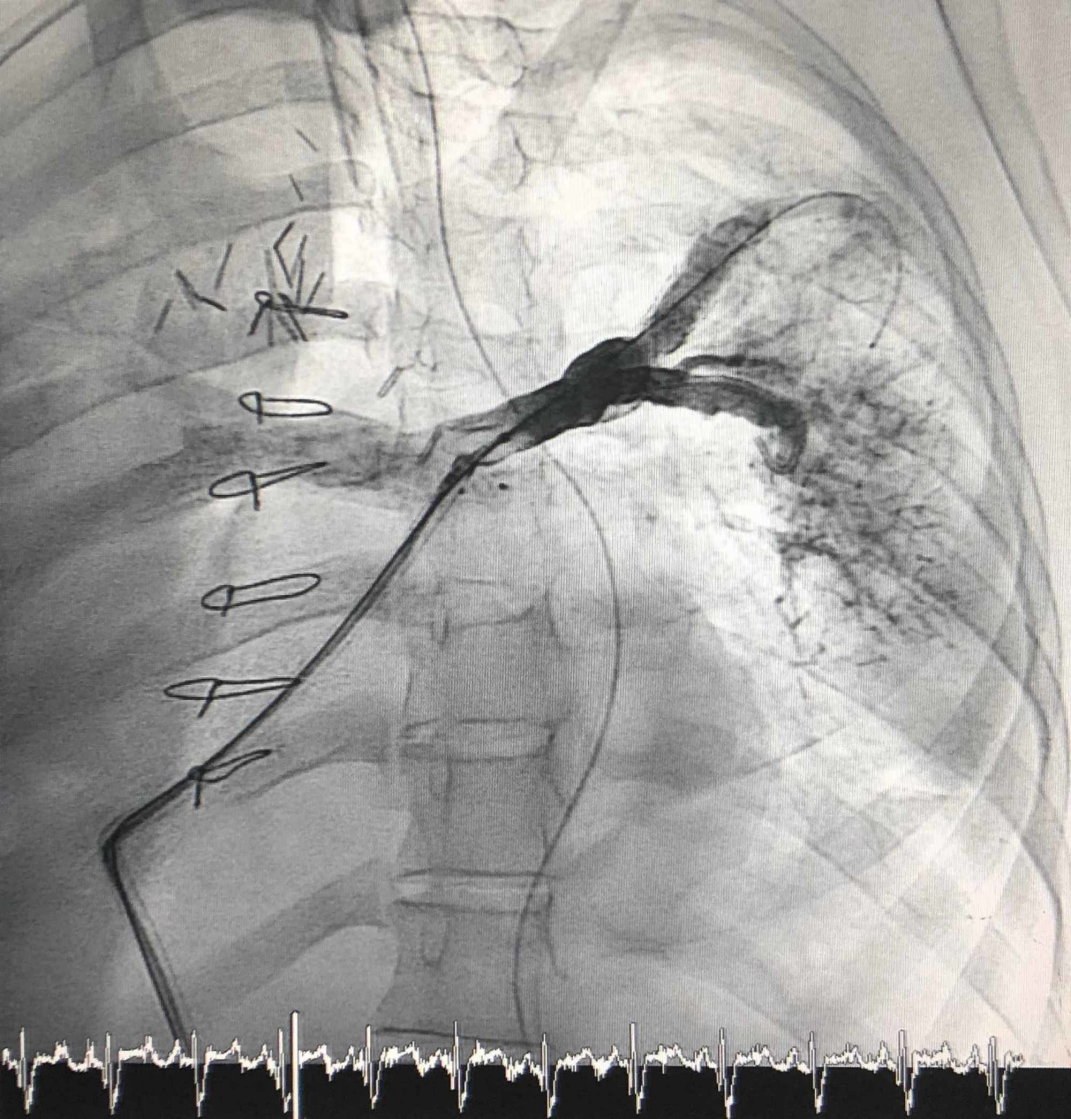
Perfusing the Left Lung Stent placement in the LPA via catheterization to increase perfusion to the left lung.

Day +266: Catheterization was performed to allow two stents to be placed and dilated in the bifurcation of the LPA (Figure 2). The patient had good blood supply to the left upper lobe, and low blood supply to the right lung and left lower lobe.

Day +300: Cardiac MRI - QP: QS 0.8. decreased RV function with a restrictive pattern.

Day +360: Cardiac catheterization was used to obtain the following measurements in room air: QP: QS 0.49; Pulmonary resistance 4.7 Wood units; measurements in 100% oxygen: QP; QS0.66, pulmonary resistance 3.6 Wood units. Pulmonary pressure 90% systemic. During the procedure, the balloon dilatation of the pulmonary arteries was performed, and a right upper lobe (RUL) stent was implanted. 

**Figure 3:**
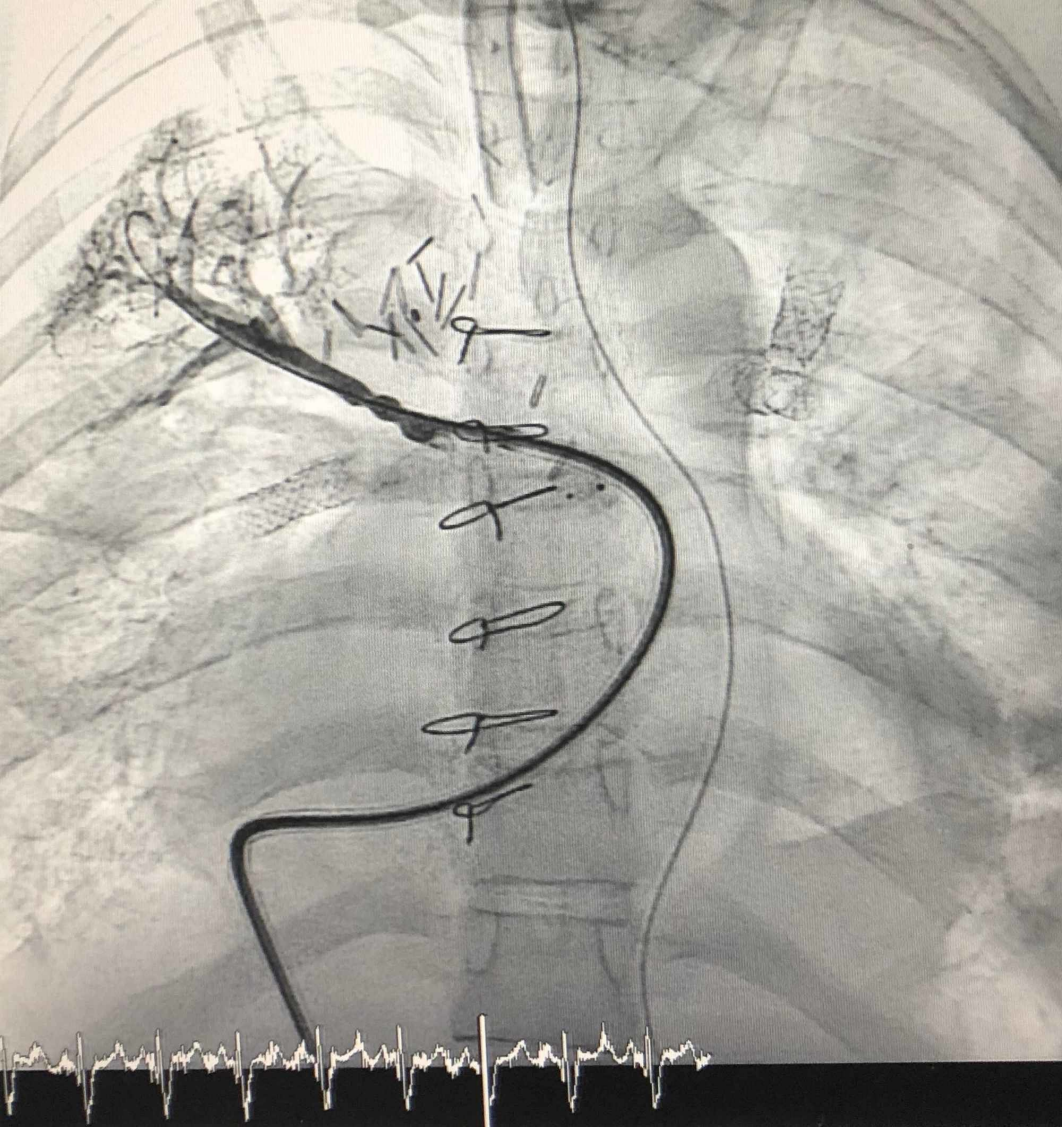
Perfusing the Right Lung Stent placement in the RPA via catheterization to increase perfusion to the RUL.

Day +422: Cardiac catheterization was used to measure a QP: QS of 0.66, a pulmonary resistance of 3.6 Wood units, and a pulmonary pressure of 90% systemic blood pressure. During catheterization, balloon dilatation of the pulmonary arteries was performed, and an RUL stent was implanted (Figure 3). 

Day +479: The patient was diagnosed with endocarditis due to Candida. This necessitated the surgical replacement of the pulmonic valve with a biological valve and resection of the vegetation. The VSD was partially closed.

Following the valve replacement surgery, patient was successfully weaned off ionotropic support and was successfully weaned from the tracheostomy with removal of the cannula and self-closure of the fistula. During her hospitalizations in intensive care unit (ICU), the patient suffered from congestive heart failure, lung congestion, peripheral edema, enlarged liver and dependence of ionotropic support. Final echocardiography demonstrated good function of pulmonic valve, small ASD with mostly a left to right shunt and pulmonary hypertension.

The patient was discharged with recommendations for PO Captopril (12.5 mg x 3/day), PO Fusid (20 mg x 2/day), PO Sildenafil (10 mg x 3/day), PO Levetiracetam (750 mg x 2/day), PO Escitalopram (5 mg x 1/day), and PO Fluconazole (400 mg x 1/day). Follow-up care by a local pediatric cardiologist was advised. The patient was instructed to maintain strict oral and dental hygiene and use antibiotic prophylaxis for dental and urogenital procedures for life following discharge. 

The decision to close the VSD in this patient was debated in multidisciplinary meetings and ultimately decided upon due to the presence of endocarditis. At the time of closure, correction was indicated by catheterization and MRI findings: The resistance of 3.6 was manageable with anti-pulmonary hypertension medication. Low QP: QS value demonstrated that the VSD was not clinically significant to maintain, and it was consequently closed to improve results.

## Discussion

A 15-year-old girl was admitted for treatment of TOF with PA/MAPCAs and bilateral peripheral pulmonary stenosis. During her hospitalization, the patient underwent several surgeries and cardiac catheterizations. The patient required prolonged hospitalization in the pediatric ICU and mechanical ventilation due to signs of heart failure. Complications warranting treatment also included Candida endocarditis and bacterial bacteremia. The patient was discharged in good condition +555 days following admission.

Surgery is required to treat TOF, with or without PA/MAPCAs definitively. The surgical course is dependent on the nature of the malformation and the severity of the symptoms. TOF without PA/MAPCAs is treated by widening the pulmonary valve and closing the VSD with a patch. Correction of these two defects will resolve the other two [2]. As such, a similar route of repair is difficult in the case of PA/MAPCAs, as the pulmonary valve cannot be widened and the blood flow from the MAPCAs to the lungs must be focused.

It is recommended that TOF with PA/MAPCAs is repaired in three stages [4]. The first of which is to promote the growth of intrapericardial pulmonary arteries and to limit blood flow to the lungs to prevent hyperperfusion. If the intrapericardial pulmonary arteries are present, this is accomplished surgically or by cardiac catheterization. In the case of hypoplastic intrapericardial pulmonary arteries, present in one-third of patients, a conduit may be placed from the right ventricle to the pulmonary arteries, or the pulmonary outflow tract may be patched while maintaining the patency of the VSD. At this point, superfluous collateral arteries may be surgically ligated or embolized during catheterization. If the collateral arteries presently assist in providing adequate arterial situation, flow will be maintained. The second stage of treatment consists of joining the multifocal sources of arterial supply into a single source, known as unifocalization of the MAPCAs. This can be done by connecting the collateral arteries to the vessels from the intrapericardial pulmonary arteries through direct anastomoses or placement of interposition grafts. The third stage of treatment is completing the anatomical repair by closing the VSD and linking the right ventricle and reconstructed pulmonary vasculature [6]. 

It is recommended that TOF with PA/MAPCAs is repaired shortly after birth, and the prognosis is worse in older patients [7]. Patients treated later in life required frequent re-intervention to maintain an acceptable right ventricular pressure. Challenges for treatment in the case of late surgery include ventricular hypoplasia, pulmonary vascular disease, loss of lung segments, and absent intra-pericardial pulmonary arteries [10]. While the cause of increased risk for older children relative to infants is not clear, it is thought to be related to the increased risk of poor nutrition and congestive heart failure, pulmonary vascular disease, and complications due to longstanding cyanosis [10]. However, repair of TOF with PA/MAPCAs in patients who did not receive early intervention is possible and displays low perioperative mortality for initial operation.

## Conclusions

TOF is a critical condition that requires surgical repair shortly after birth. Delayed intervention to repair the cardiac defects increases the risk of complications and mortality in TOF patients. As such, this paper observed the course of treatment for a 15-year-old patient with TOF in its most severe form, with concurrent PA and MAPCAs. Due to the age of the patient at the time of admission, the turbulent course of treatment may have been tempered if the patient had been treated sooner. However, due to the arborization of MAPCAs and bilateral peripheral pulmonary stenosis in this patient, the treatment of this patient would likely have been difficult at any age. 
